# The Multidimensional Impact of Traditional Orthopaedic Casting and the Role of Emerging Immobilization Technologies: A Narrative Review

**DOI:** 10.3390/healthcare14142039

**Published:** 2026-07-08

**Authors:** James Stavitz, Ryan Porcelli, Aatmaja Vachhani

**Affiliations:** 1 Department of Athletic Training Education, College of Health Professions and Human Services, Kean University, 1000 Morris Avenue, Union, NJ 07083, USA; 2 School of Interwoven Arts and Sciences, Krea University, Sri City Campus, 5655, Central Expressway, Chittoor 517646, Andhra Pradesh, India

**Keywords:** orthopaedic casting, fracture immobilization, light-cured polymer mesh, 3D-printed casts, patient-centered care, immobilization complications

## Abstract

Background: Traditional orthopaedic casting has remained the cornerstone of non-surgical fracture management for more than a century. Although plaster and fiberglass casts reliably stabilize fractures, they are associated with physical, psychological, emotional, social, and economic burdens that extend beyond bone healing. Children, older adults, and individuals with pre-existing vulnerabilities may be disproportionately affected. Despite increasing recognition of these complications, existing orthopaedic literature has historically prioritized radiographic healing and biomechanical stability, with limited synthesis of the broader multidimensional patient impact of traditional casting. Emerging technologies such as light-cured polymer mesh (LCPM) systems and 3D-printed lattice immobilizers have been developed to address these limitations and better align fracture care with patient-centered principles. Methods: A narrative review was conducted using a structured and transparent literature identification approach informed by PRISMA reporting principles; however, this study was not conducted as a formal systematic review and did not include risk-of-bias assessment or quantitative synthesis. A broad search of PubMed, Scopus, Web of Science, and Google Scholar was performed for studies published between January 2000 and July 2025. Search strategies combined MeSH terms and free-text keywords relating to orthopaedic casting, complications, psychosocial impacts, LCPM, and 3D-printed immobilizers. Following duplicate removal and a structured review process, 87 studies were included in the final narrative synthesis. Eligible studies included randomized controlled trials, observational studies, qualitative research, case series, and systematic reviews. Data were synthesized narratively across five domains: physical, psychological, emotional/social, economic, and technological alternatives. Results: Traditional plaster and fiberglass casts were consistently associated with musculoskeletal deterioration, joint stiffness, dermatological complications, and hygiene challenges. Psychological and emotional consequences included cast-induced anxiety, claustrophobia, depressive symptoms, and diminished autonomy. Social participation was frequently reduced due to mobility restrictions and perceived stigma, while economic impacts included hidden out-of-pocket expenses, caregiver burden, lost wages, and disparities in access to follow-up care. In contrast, emerging alternatives demonstrated promising advantages. LCPM systems improved ventilation, comfort, and hygiene, while reducing saw-related anxiety. Preliminary evidence suggests that both LCPM and 3D-printed systems may support improved patient experience and earlier return to selected activities, although larger comparative studies are needed to confirm effects on complication rates and long-term outcomes. Over time, these benefits may help offset higher upfront material costs. Conclusions: Fracture care should be evaluated not only by radiographic healing but also by patient-centered outcomes such as comfort, independence, and quality of life. Traditional casting imposes significant multidimensional burdens, whereas newer technologies such as LCPM and 3D-printed systems may offer a more holistic approach to immobilization while maintaining acceptable fracture stability in appropriately selected patient populations. While current evidence indicates potential physical, psychological, and economic advantages, large-scale comparative trials remain necessary to confirm long-term clinical, psychosocial, and cost-effectiveness outcomes across diverse populations. Future integration of emerging immobilization technologies into clinical practice may support more patient-centered, function-oriented, and cost-conscious approaches to fracture care.

## 1. Introduction

Orthopaedic casting has remained the primary non-surgical approach for fracture stabilization for more than a century. Since the introduction of Plaster of Paris (PoP) in the 19th century and the later adoption of fiberglass, casts have been central to emergency and outpatient trauma care. These casts are widely used because they stabilize fractures and support healing. Union rates for non-displaced extremity fractures treated conservatively are often reported above 90%. This is when proper immobilization protocols are followed [1,2]. Fractures lead to millions of healthcare visits each year. Many patients also experience complications related to immobilization. These issues have been estimated to affect about 15–30% of patients [3]. However, the benefits of traditional casting are accompanied by significant drawbacks that extend far beyond bone union. Physical complications such as joint stiffness, muscle atrophy, skin irritation, and pressure-related pain are frequently reported [3]. Muscle atrophy can occur quickly during immobilization. Reductions in muscle size have been observed within the first 1–2 weeks of casting. Strength losses can approach 20–30% with prolonged immobilization. Joint stiffness and range-of-motion deficits are common after cast removal. These issues have been reported in approximately 25–40% of patients and are more likely when immobilization lasts longer than four weeks. The extent of immobilization and inclusion of adjacent joints may substantially influence the severity of post-immobilization stiffness and functional limitation [3]. More restrictive immobilization constructs involving multiple joints are generally associated with greater range-of-motion limitation and delayed functional recovery compared with less restrictive approaches [3]. However, reporting regarding immobilization configuration and joint inclusion remains inconsistent across the available literature, limiting direct comparison between studies. Skin-related complications are also reported. These include maceration, irritation, and pressure injuries. They occur in about 5–15% of patients and are more common in pediatric and older populations [3].

Immobilization can also place psychological strain on patients, contributing to anxiety, claustrophobia, and loss of autonomy [4]. Some patients do not tolerate casts well. Anxiety and claustrophobic responses have been reported in about 20–30% of patients. These symptoms are often worse in pediatric populations, especially during immobilization and cast removal. Emotional challenges, such as frustration, dependency, and reduced self-esteem, further complicate recovery, particularly in cases requiring immobilization beyond six weeks [5]. Casting also imposes social and economic burdens. These include absence from work or school, caregiver stress, and unplanned financial strain [6]. Time away from work is common after fracture immobilization. Absences typically range from 2 to 6 weeks in non-manual jobs and may be longer in physically demanding roles. Caregivers often provide several additional hours of support each day during early recovery. These consequences are particularly pronounced in vulnerable populations. Children, older adults, and individuals with limited socioeconomic resources are often most affected. Despite these well-documented challenges, most orthopaedic research has historically prioritized radiographic healing and biomechanical stability [7,8,9]. As a result, the broader psychosocial and economic dimensions of immobilization are often overlooked. This gap in the literature highlights the need for a more comprehensive evaluation of fracture immobilization that incorporates both clinical and patient-centered perspectives.

Emerging immobilization technologies differ substantially from traditional plaster and fiberglass casting in both design and application. Light-cured polymer mesh (LCPM) systems consist of flexible polymer-based mesh materials that are molded directly to the extremity and subsequently rigidized through light-activated curing [1,3]. Unlike traditional circumferential casts, these systems utilize ventilated lattice-style architectures designed to improve airflow, reduce moisture accumulation, decrease overall weight, and enhance patient comfort while maintaining fracture stability [1,3]. Similarly, 3D-printed immobilizers use digital scanning and additive manufacturing techniques to create anatomically contoured orthoses with customizable lattice structures intended to improve fit, hygiene, and usability during recovery [1,3]. These technologies have emerged as patient-centered alternatives that aim to address many of the physical and psychosocial limitations associated with conventional casting approaches [1,3].

Early investigations of these emerging immobilization technologies suggest that they may preserve fracture stability while mitigating several limitations associated with traditional plaster and fiberglass casting [3]. Light-cured polymer mesh (LCPM) systems (e.g., FlexiOH^®^; OrthoHeal Inc., Coppell, TX, USA) and 3D-printed lattice casts are newer alternatives. These lightweight, breathable, and washable designs may improve hygiene, enhance comfort, and support social participation during recovery [3]. Early studies on polymer mesh and 3D-printed immobilization systems suggest high levels of patient satisfaction and favorable patient-reported experiences; however, direct comparative evidence remains limited and additional large-scale investigations are needed to confirm these observations [1,3]. Emerging materials and designs have also been developed to address common complications associated with conventional casting, including skin irritation and moisture-related issues [3]. In addition, their lightweight and breathable structure may support improved functional tolerance and patient experience during immobilization [1].

Meaningful direct comparison among traditional plaster and fiberglass casts, light-cured polymer mesh (LCPM) systems, and 3D-printed immobilizers remains challenging within the current evidence base. Available studies differ substantially with respect to fracture location, patient demographics, duration of immobilization, outcome measures, follow-up periods, and study design. In addition, many investigations involving emerging technologies consist of feasibility studies, pilot cohorts, or observational reports rather than large-scale randomized comparative trials [1,3,4]. Consequently, existing evidence more consistently supports comparisons of patient-centered experiences and selected functional outcomes than definitive conclusions regarding comparative clinical superiority or long-term effectiveness, reinforcing the need for a multidimensional perspective on fracture management.

Despite growing recognition of the physical and psychosocial consequences of fracture immobilization, existing reviews have largely focused on biomechanical outcomes, radiographic healing, or the performance of individual immobilization materials [1,2,3,4]. To our knowledge, no prior review has comprehensively synthesized the interconnected physical, psychological, emotional, social, and economic impacts of traditional orthopaedic casting while simultaneously evaluating emerging technologies through a patient-centered framework [1,2,3,4]. Given increasing emphasis on quality of life, shared decision-making, and value-based care within modern orthopaedics [5], a broader multidimensional perspective is needed to better inform clinicians, patients, and future research efforts. Accordingly, this narrative review was undertaken to address this gap by integrating evidence across multiple domains and examining the potential role of emerging immobilization strategies in supporting more holistic fracture care.

**Figure 1:**
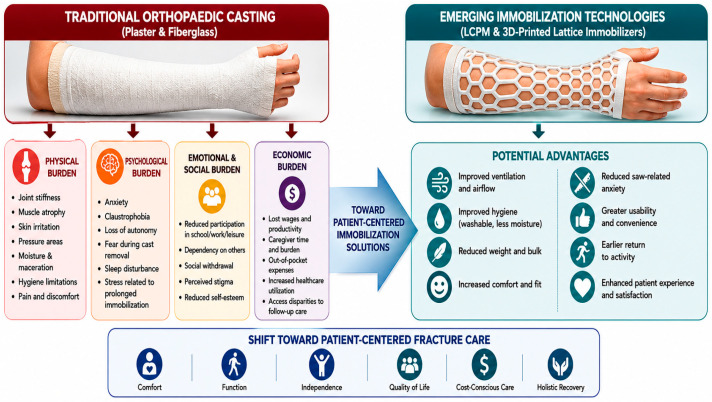
Conceptual overview of the multidimensional burden associated with traditional orthopaedic casting and the proposed advantages of emerging immobilization technologies. Traditional plaster and fiberglass casts may contribute to physical, psychological, emotional/social, and economic burdens that extend beyond fracture stabilization alone. Emerging technologies such as light-cured polymer mesh (LCPM) systems and 3D-printed lattice immobilizers have been proposed as potential approaches to address some of these limitations while supporting a more patient-centered model of fracture care. The advantages illustrated are conceptual and should not be interpreted as evidence of definitive clinical superiority over traditional casting methods.

A conceptual overview of the multidimensional burden associated with traditional orthopaedic casting and the proposed advantages of emerging immobilization technologies is presented in Figure 1. This narrative review synthesizes the multidimensional consequences of traditional casting across physical, psychological, emotional, social, and economic domains. It also evaluates emerging alternatives that aim to better align fracture care with patient-centered principles. Given the heterogeneity of the available evidence, the purpose of this review was not to establish definitive comparative superiority between immobilization systems, but rather to synthesize the current literature regarding the multidimensional patient impact of traditional casting and the emerging role of alternative immobilization technologies within patient-centered fracture care.

Figure 1 Conceptual Overview of the Multidimensional Burden of Traditional Orthopaedic Casting and the Potential Advantages of Emerging Immobilization Technologies.

## 2. Methodology

This narrative review was conducted to examine the impacts of traditional orthopaedic casting. We focused on multiple domains, including physical, psychological, emotional, social, and economic effects. We also evaluated emerging alternatives, with a focus on the LCPM system. The goal was not only to summarize existing evidence. We also sought to synthesize and interpret how casting influences patient outcomes across physical, psychological, emotional, social, and economic domains. In framing this review, we aimed to bridge clinical practice with patient-centered considerations. This approach provides a broader perspective on immobilization than is typically emphasized in orthopaedic literature.

Although this review does not constitute a systematic review, a structured and transparent approach to literature identification and synthesis was used to enhance rigor. Reporting was guided by PRISMA principles to enhance transparency and clarity. However, this study was not conducted as a formal systematic review, and key components such as risk-of-bias assessment and quantitative synthesis were not performed. Accordingly, findings should be interpreted as a structured narrative synthesis [10]. Due to substantial heterogeneity across the available literature, including differences in fracture location, immobilization techniques, patient demographics, duration of immobilization, outcome measures, follow-up periods, and study design, a formal meta-analysis was not considered methodologically appropriate. Additionally, many available studies consisted of observational investigations, pilot studies, case series, narrative reviews, and heterogeneous patient populations that limited direct quantitative comparison. Accordingly, a narrative review methodology was selected to allow broader synthesis of the multidimensional physical, psychological, emotional, social, and economic consequences associated with traditional casting and emerging immobilization technologies. This structured approach allowed documentation of the search strategy and transparent application of inclusion and exclusion criteria. We placed emphasis on including both quantitative and qualitative evidence. This approach helped ensure that the multidimensional impacts of casting were adequately represented.

To improve comprehensiveness, we prioritized studies with direct clinical relevance. We also included patient-reported experiences when available. This helped provide a more complete understanding of outcomes [11]. Qualitative accounts, case studies, and patient satisfaction surveys were included alongside randomized and observational studies. This approach reflects an effort to better capture the full patient experience [12]. This hybrid approach works well for studying fracture care. Psychosocial outcomes are not always captured in large trials. However, they are still very important in clinical practice.

### 2.1. Literature Search Strategy

A broad literature search was conducted across four major databases. These included PubMed, Scopus, Web of Science, and Google Scholar. Studies published between January 2000 and July 2025 were included [13]. The decision to begin in 2000 was made to capture contemporary evidence reflective of current clinical practice while ensuring two decades of coverage. The search strategy used both Medical Subject Headings (MeSH) and free-text keywords [14]. Boolean operators were also applied. This helped improve search sensitivity while still maintaining specificity [11,12,13]. Representative search strings included: (“orthopaedic cast” OR “plaster cast” OR “fiberglass cast”) AND (“complications” OR “adverse effects” OR “immobilization”); (“FlexiOH” OR “light-cured polymer cast” OR “hybrid mesh cast”); (“3D-printed cast” OR “3D printing” AND “fracture immobilization”); and (“patient-centered care” AND “fracture” AND “casting”). Additional keywords such as “skin irritation,” “muscle atrophy,” “caregiver burden,” and “quality of life” were applied in secondary searches to support coverage of patient-reported outcomes and economic impacts.

To improve coverage, citation searches were also performed. Both backward and forward searches of key articles were included. This helped identify studies that were not found in the initial database search [11,12,13]. Grey literature was also reviewed to minimize publication bias [15]. This included conference proceedings, theses, and government or policy reports relevant to novel immobilization technologies. All identified references were imported into EndNote (Clarivate Analytics) for management, and duplicates were removed prior to screening [16].

The literature search identified 1472 records. After removal of duplicate records, 833 unique publications remained for further review. Following title and abstract review, 214 articles were selected for full-text assessment. Of these, 87 studies met all eligibility criteria and were included in the final synthesis. The final evidence base included a range of study designs. These included randomized controlled trials, cohort studies, case–control studies, and cross-sectional surveys. It also included case series, systematic reviews, and qualitative studies, which allowed both clinical outcomes and patient experiences to be captured.

### 2.2. Study Selection

The identified articles were reviewed at the title and abstract level to assess relevance to the aims of this narrative review [11]. Studies were excluded at this stage if they were clearly irrelevant to orthopaedic casting or immobilization (e.g., dental applications, veterinary studies, or unrelated biomaterial research). This process excluded 619 records, leaving 214 studies for full-text assessment.

**Figure 2:**
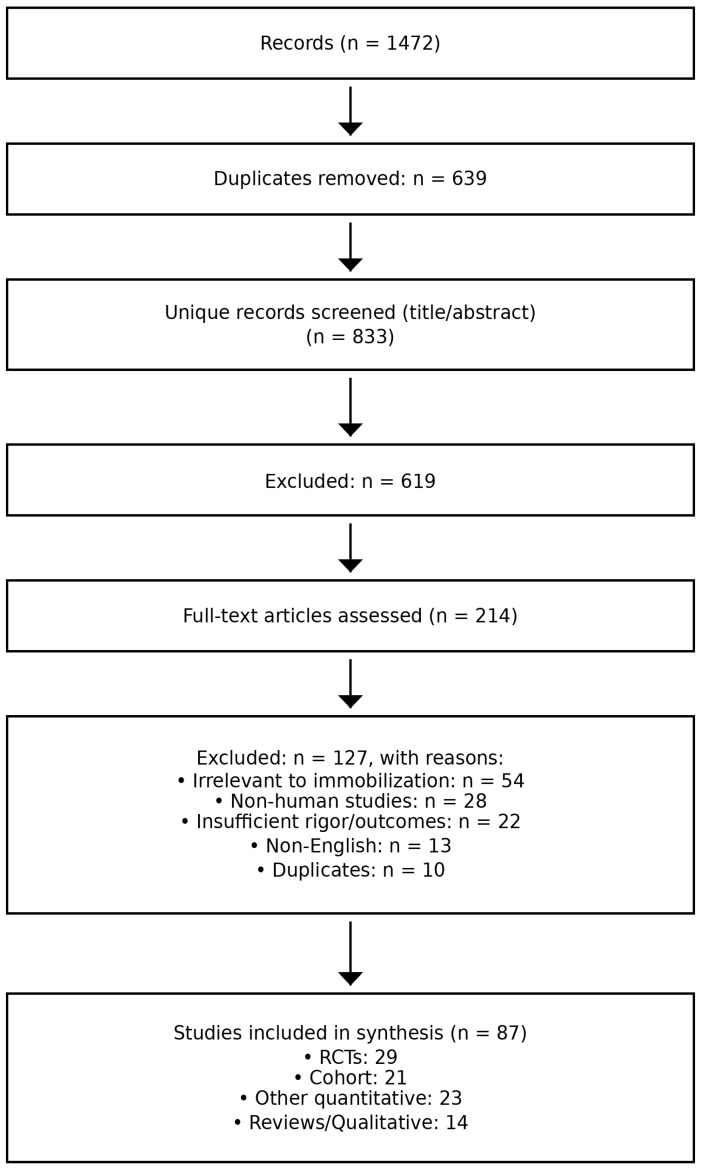
Illustrates the structured literature identification, screening, eligibility assessment, and study inclusion process used in this narrative review. Database searches were conducted across PubMed, Scopus, Web of Science, and Google Scholar. Following duplicate removal and application of predefined inclusion and exclusion criteria, 87 studies were included in the final narrative synthesis.

At the full-text stage, studies were excluded for several reasons. Some studies were not directly related to fracture care or immobilization (*n* = 54). Others were non-human or cadaveric (*n* = 28), had poor methodology or insufficient outcome reporting (*n* = 22), were not published in English (*n* = 13), or represented duplicate records identified during full-text review (*n* = 10). After applying these exclusions, a total of 87 studies were included in the final synthesis (Figure 2).

Figure 2 PRISMA-Informed Literature Identification and Study Selection Process.

Of the included studies, 29 were randomized controlled trials, 21 were cohort studies (prospective or retrospective), 12 were case–control or cross-sectional designs, 11 were case series or clinical reports, 7 were systematic or narrative reviews, and 7 were qualitative or mixed-methods studies exploring patient-reported outcomes and caregiver perspectives. This range of study designs allowed for a broad evaluation of clinical outcomes. It also allowed assessment of patient-centered impacts related to traditional casting and its alternatives. To support consistency in study selection, included articles were reviewed iteratively by the author team with decisions resolved through discussion [11].

### 2.3. Inclusion and Exclusion Criteria

Studies were included if they involved human participants with fractures. All ages were considered. Studies also had to be published in peer-reviewed journals in English between January 2000 and July 2025 [11,17,18]. To align with the multidimensional aims of this review, eligible studies were required to address at least one of the following domains: physical impacts (e.g., skin complications, muscle atrophy, or durability issues), psychological impacts (e.g., cast-induced anxiety, claustrophobia, or depressive symptoms), emotional or social impacts (e.g., autonomy, caregiver burden, or social participation), economic impacts (e.g., direct or indirect costs, lost wages, or household financial strain), or evaluation of emerging alternatives such as the LCPM system or 3D-printed lattice immobilizers. Both interventional and observational designs were considered, and qualitative or mixed-methods studies were included when they provided relevant patient-reported outcomes or caregiver perspectives. Case reports and case series were also eligible if they offered unique or clinically meaningful insights into complications or experiences not widely represented in larger trials [19].

Exclusion criteria were applied to maintain focus and relevance to the aims of the review [11,18]. Studies were excluded if they involved non-human models (animal or cadaveric work) or in vitro biomechanical simulations without patient outcomes. Conference abstracts, editorials, letters to the editor, and commentaries were excluded unless they presented original data of sufficient quality. Publications in languages other than English were excluded due to resource limitations in translation and validation. In addition, studies unrelated to fracture immobilization, such as those focused exclusively on dental, veterinary, or prosthetic casting, were excluded. Finally, studies lacking sufficient methodological detail or outcome reporting were excluded during full-text review.

### 2.4. Screening and Data Extraction

As described, the review process involved iterative evaluation of titles, abstracts, and full texts to determine relevance to the aims of this narrative review. Studies meeting inclusion requirements, or those deemed potentially relevant, were advanced to full-text review. At both stages, differences in interpretation were resolved through discussion.

Extracted information was reviewed for consistency and accuracy before synthesis [20]. Extracted variables included bibliographic details such as author, year, and journal. Additional data included study design, geographic location, sample size, participant demographics, type of immobilization, duration of immobilization, and comparator groups where applicable. Outcomes of interest were classified into five predefined domains: (1) physical (e.g., skin integrity, stiffness, muscle atrophy); (2) psychological (e.g., anxiety, claustrophobia, depressive symptoms); (3) emotional and social (e.g., autonomy, caregiver burden, participation restrictions); (4) economic (e.g., direct costs, indirect costs, healthcare utilization); and (5) technological alternatives (e.g., patient-reported outcomes related to LCPM or 3D-printed systems). Where available, qualitative findings, including patient narratives, caregiver perspectives, and satisfaction metrics, were extracted to supplement clinical outcome data.

Extracted data were reviewed for consistency and accuracy prior to synthesis [21]. Any discrepancies were resolved by re-examining the original study reports. Studies with incomplete or unclear reporting were not excluded but were noted and interpreted cautiously within the synthesis. Data were synthesized narratively [22], emphasizing recurring patterns, common complications, and emerging themes relevant to both traditional casting and modern immobilization technologies.

### 2.5. Quality Appraisal

Although this review was conducted narratively rather than systematically, efforts were made to maintain methodological rigor and transparency [11,12,13]. Each study included was appraised according to design, sample size, methodological clarity, and reporting quality. Randomized controlled trials (RCTs) and prospective cohort studies were assigned the greatest evidentiary weight given their capacity for causal inference and control of confounders [23]. Retrospective cohort studies, cross-sectional analyses, and case series were classified as moderate in evidentiary value, while single case reports were included primarily for illustrative or hypothesis-generating purposes [24]. Qualitative studies and patient-reported outcome investigations were retained when they offered unique insights into psychological, emotional, or social experiences not otherwise captured in quantitative data [25].

Study quality and reporting clarity were considered during synthesis using general principles drawn from established appraisal frameworks [26,27,28]. Domains considered included selection bias, reporting bias, appropriateness of outcome measures, attrition and follow-up, and transparency of analytic methods. While no numerical scoring system was applied, studies judged to have substantial methodological limitations (e.g., incomplete follow-up, small sample size without justification, or selective reporting) were weighted less heavily in the synthesis.

### 2.6. Synthesis Approach

Findings were synthesized narratively, with the aim of identifying recurring patterns, cross-cutting themes, and areas of divergence across the included literature. To facilitate a multidimensional appraisal, results were grouped into five predefined domains: (1) physical impacts, (2) psychological impacts, (3) emotional and social impacts, (4) economic impacts, and (5) emerging technological alternatives. This structure allowed integration of heterogeneous evidence, making sure that both clinical outcomes and patient-centered perspectives were represented.

Emerging immobilization alternatives, including the LCPM system and 3D-printed lattice casts, were discussed separately to highlight their capacity to mitigate the limitations of plaster and fiberglass [1]. Within this synthesis, priority was given to triangulating findings across quantitative outcomes (e.g., complication rates, time to return to work, healthcare utilization) and qualitative evidence (e.g., patient narratives, caregiver perspectives, satisfaction reports) [29]. This hybrid integration approach maintained that conclusions reflected not only radiographic or biomechanical outcomes but also the psychosocial and economic dimensions of fracture care. Thematic synthesis also enabled identification of gaps in the literature, providing direction for future research [30]. Given the broad scope of the available literature, fracture populations, anatomical regions, immobilization strategies, and patient demographic characteristics varied substantially across included studies. Variables such as fracture location, adjacent joint immobilization, patient age, comorbidities, duration of immobilization, and socioeconomic context may independently influence both complication rates and patient-reported outcomes. Accordingly, findings were interpreted within the context of this heterogeneity, and direct comparisons between traditional casting, LCPM systems, and 3D-printed immobilization approaches should be interpreted cautiously.

## 3. Results

### 3.1. Physical Impacts of Traditional Orthopaedic Casting

Traditional orthopaedic casting remains the most widely used method of fracture immobilization [3], but its physical drawbacks are well documented and extend beyond the immediate stabilization of bone [31]. Prolonged immobilization leads to predictable musculoskeletal deterioration [32]. Joint stiffness arises from reduced synovial fluid circulation and periarticular contracture. Muscle atrophy can develop within the first week of casting and may progress rapidly. Both experimental and clinical studies show measurable muscle loss during immobilization. Muscle cross-sectional area can decrease by about 3–10% within the first 7–14 days. Strength losses may reach 20–30% with longer casting durations. This occurs particularly in older adults or those with pre-existing mobility limitations [33]. These changes often delay rehabilitation and increase the risk of long-term functional deficits [31,32,33]. Post-immobilization range-of-motion deficits have been reported in approximately 25–40% of patients following cast removal, particularly when immobilization exceeds four weeks.

The cast itself can also be a direct source of physical discomfort [34]. Improper padding, swelling beneath the rigid material, or uneven pressure distribution frequently cause localized pain and stiffness. Although uncommon, severe complications of casting may include neurovascular compromise and acute compartment syndrome [35]. Acute compartment syndrome related to casting is rare. It has been reported in about 0.1–0.3% of cases. This is more likely when swelling occurs under circumferential casts. Skin health is another recurring concern [36]. The enclosed environment fosters moisture retention, odor, and irritation, and in warm or humid climates, maceration and secondary infection become more likely [37]. Skin complications are commonly reported with casting. These include maceration, pressure ulcers, contact dermatitis, and superficial infections. They occur in about 5–15% of patients, but rates can exceed 20% in pediatric hip spica casting. Children are particularly vulnerable, as their more delicate skin and limited ability to articulate discomfort can allow complications to progress before detection [38].

Durability issues compound these challenges. Plaster casts, though inexpensive, are prone to cracking or deformation if not fully cured or reinforced [39], while fiberglass, though stronger, may still weaken or loosen if improperly applied [40]. Some studies have reported issues with cast durability. Breakdown or structural problems occur in about 4–12% of plaster casts and 2–5% of fiberglass casts. These cases often require reinforcement or full reapplication. These failures often require reapplication, prolong immobilization, and expose patients to additional inconvenience, cost, and risk. Unplanned cast replacement has been associated with immobilization extensions averaging 7–14 additional days in fracture management cohorts.

Certain populations bear a disproportionate burden of these complications. Older adults, individuals with chronic conditions such as diabetes or arthritis, and those with reduced vascular health face an increased risk of falls, pressure injuries, venous thromboembolism, and delayed recovery [41]. Older adults who are immobilized have higher fall rates. These rates are about 1.5–2.0 times higher than those who are not immobilized. Pressure injuries during lower-extremity casting have also been reported in about 8–14% of geriatric patients. For these groups, the physical effects of casting often extend beyond the fracture itself and may threaten overall health and independence [42]. Additionally, venous thromboembolism risk during lower-limb immobilization has been estimated between 2–6%, particularly in patients with pre-existing vascular or metabolic comorbidities.

Direct comparison between traditional plaster and fiberglass casts and emerging immobilization technologies remains challenging within the current evidence base. Existing studies differ substantially with respect to fracture location, patient demographics, duration of immobilization, outcome measures, and follow-up periods, limiting rigorous comparative interpretation across physical domains [3]. Consequently, current evidence more reliably supports observations regarding patient experiences and selected functional considerations than definitive conclusions about comparative effectiveness, complication reduction, or long-term outcomes among immobilization strategies.

Although traditional plaster and fiberglass casts remain highly effective for fracture stabilization, the evidence consistently demonstrates that immobilization-related complications extend beyond bone healing alone. Musculoskeletal deterioration, dermatologic complications, and vascular concerns collectively influence functional recovery and may contribute to subsequent psychological and social burdens, reinforcing the need for more patient-centered approaches to fracture care [3,43].

### 3.2. Psychological Impacts of Traditional Orthopaedic Casting

The physical discomfort and functional restrictions associated with casting are frequently accompanied by psychological distress [44]. Cast-induced anxiety and claustrophobia have been increasingly reported, with patients describing feelings of confinement, restlessness, and loss of control within days of immobilization [45]. Studies have reported anxiety in immobilized patients. Clinically meaningful anxiety symptoms occur in about 25–40% of cases. Claustrophobic distress is reported in about 10–20% of patients with circumferential casts. In some cases, these symptoms can become more severe. Patients may experience panic episodes or request early cast removal. This has been reported in about 3–8% of patients, especially in younger individuals and those with anxiety disorders [46].

Cast removal represents an additional source of psychological stress [47]. Oscillating saws are commonly used for cast removal. They produce loud noise, vibration, and unfamiliar sensations. These factors are associated with fear in both children and adults, especially when preparation is limited [48]. Fear during cast removal is common in children. Studies report fear responses in about 40–60% of pediatric patients. This is often accompanied by increased heart rate and behavioral distress. In adult populations, anticipatory anxiety related to cast removal has been reported in approximately 15–25% of patients, particularly during first-time immobilization experiences [49].

Prolonged immobilization is also associated with sustained psychological strain [50]. Disruption of daily routines, increased dependence on caregivers, and limitations in performing routine tasks are associated with reductions in perceived independence [51]. Depressive symptoms have been reported during prolonged casting. Rates range from about 15–30%. These rates are higher when immobilization lasts longer than six weeks. Among individuals with pre-existing mental health conditions or limited social support, reported prevalence may exceed 40% [52].

Psychological responses vary across age groups [44]. Adolescents have reported embarrassment and social withdrawal related to visible casting, with prevalence estimates ranging from approximately 30–45% in fracture cohorts. In older adults, concerns regarding loss of independence and delayed recovery are commonly reported [53]. In pediatric populations, caregiver-reported behavioral distress, including irritability and sleep disruption, has been documented in approximately 35–55% of cases, with sleep disturbances specifically reported in 20–30% during early immobilization [54].

Collectively, these findings suggest that the psychological consequences of immobilization extend beyond transient discomfort and may influence broader emotional well-being, social engagement, and recovery experiences. Individual coping strategies and support systems appear to moderate these effects, highlighting the importance of patient-centered approaches that address both physical and psychological needs during fracture management [55,56,57].

### 3.3. Emotional and Social Impacts of Traditional Orthopaedic Casting

The psychological effects of immobilization are frequently accompanied by broader emotional and social changes that influence daily functioning and self-perception [58]. Feelings of helplessness, frustration, and reduced self-worth have been reported in association with loss of independence during recovery [59]. Activities such as bathing, dressing, and managing household responsibilities may become more difficult, particularly during the early phases of immobilization. Many patients experience temporary dependence during casting. This often affects at least one activity of daily living. This has been reported in about 45–70% of patients within the first 2–4 weeks [60]. Health-related quality-of-life assessments, including SF-36 and EQ-5D measures, have demonstrated short-term reductions of 20–40% in role-physical and social-functioning domains during immobilization periods.

These challenges often extend to family members and caregivers. Patients have reported feelings of guilt related to increased reliance on others, while caregivers may experience fatigue, anxiety, and role strain [61]. Caregiver burden is also common. Moderate-to-high burden has been reported in about 30–50% of informal caregivers. This is especially seen in pediatric and geriatric cases. Disruptions in household routines, including missed workdays and redistribution of daily responsibilities, have been documented in more than 60% of families in pediatric casting populations [62].

Reductions in social participation are also commonly reported. Patients may limit engagement in public or recreational activities due to mobility restrictions, fatigue, or concerns related to appearance [63]. Social participation often decreases during immobilization. Studies report reduced activity in about 40–65% of patients. This occurs during active casting periods. In pediatric populations, school absenteeism ranging from 5 to 15 days has been reported during early immobilization, with continued limitations in extracurricular participation beyond this period [64]. Among older adults, reductions in community engagement of approximately 30–50% have been reported during lower-extremity immobilization, often associated with concerns about fall risk.

Workplace disruption represents an additional dimension of social impact. Time away from work is common after fracture immobilization. Absences are about 2–6 weeks for non-manual jobs. They can extend to 6–12 weeks for physically demanding roles [65]. In some cases, delayed return to work has been associated with ongoing emotional fatigue, reduced confidence, or residual functional limitations [66]. Studies have also identified reduced workplace productivity in approximately 20–35% of patients during the first three months following cast removal.

Despite these challenges, adaptive responses and resilience have also been reported, particularly among patients with strong social support networks. These findings suggest that the social environment surrounding fracture recovery may substantially influence emotional well-being, treatment adherence, and overall rehabilitation outcomes. Opportunities for meaningful engagement, caregiver support, and preservation of autonomy may therefore represent important yet underappreciated components of patient-centered fracture management [67,68].

### 3.4. Economic Impacts of Traditional Orthopaedic Casting

The emotional and social consequences of immobilization are frequently accompanied by financial burdens affecting patients, caregivers, and healthcare systems [69]. Traditional plaster and fiberglass casts are often considered cost-effective due to relatively low material and application costs, typically ranging from approximately $50–$150 per application depending on the clinical setting [70]. However, this estimate does not account for broader direct and indirect costs associated with fracture care [71]. Total episode-of-care costs, including follow-up visits, imaging, cast changes, and rehabilitation, have been reported to range from several hundred to several thousand U.S. dollars, with higher cumulative costs observed in lower-extremity fractures.

Patients commonly incur additional out-of-pocket expenses, including costs related to cast protection, mobility aids, hygiene products, and assistance with daily activities [72]. Studies have reported additional household expenditures ranging from approximately $100 to over $1000 during the immobilization period, particularly when assistive devices or transportation services are required. In lower-income populations, fracture-related expenses have been reported to account for 10–30% of monthly household income in some settings [73]. Financial constraints have also been associated with missed follow-up appointments, which have been reported in approximately 5–15% of orthopaedic trauma cohorts [71].

Indirect costs further contribute to the economic burden. These costs, including lost productivity and informal caregiving, may account for approximately 40–60% of the total economic burden associated with extremity fractures [74]. Lost wages and reduced productivity are commonly reported, particularly among individuals in physically demanding occupations or those who are self-employed [61,66,71]. Return-to-work studies have estimated productivity losses equivalent to approximately 2–12 weeks of wages, depending on injury severity and occupational demands.

Caregivers may also experience financial strain. Time spent providing assistance has been estimated at approximately 2–6 additional hours per day during early recovery, with total caregiving time ranging from 40 to over 150 h during a typical 6-week immobilization period [75]. Associated costs, including transportation, equipment, and childcare adjustments, contribute to the overall economic impact [76]. Valuation studies have estimated that informal caregiving may represent costs ranging from several hundred to several thousand U.S. dollars per fracture episode, depending on injury severity and duration of care [77].

Economic disparities influence access to care and recovery trajectories. Patients in rural or underserved areas may face barriers to follow-up care, rehabilitation services, and access to necessary medical supplies [61,71,78]. Delayed follow-up has been reported more frequently among patients residing greater distances from care facilities. In some cases, access barriers have been associated with non-adherence to treatment recommendations, including unsupervised cast removal, with corresponding increases in complication risk [61,71,78].

Collectively, these findings demonstrate that the economic burden of fracture immobilization extends far beyond the initial cost of cast application. Lost productivity, caregiver demands, transportation expenses, and disparities in access to follow-up care may substantially influence recovery trajectories and treatment adherence, particularly among vulnerable populations. These observations reinforce the importance of considering economic and social determinants of health when evaluating fracture management strategies and highlight the potential value of technologies that reduce downstream complications and indirect costs. The multidimensional impacts of traditional orthopaedic casting across physical, psychological, emotional, social, and economic domains are summarized across domains in Table 1.

Taken together, the findings synthesized across physical, psychological, emotional, social, and economic domains demonstrate that the impact of traditional orthopaedic casting extends well beyond fracture stabilization alone. These domains appear highly interconnected, as physical discomfort and functional limitations may contribute to psychological distress, reduced social participation, caregiver burden, and downstream financial consequences. This multidimensional perspective suggests that successful fracture management should be evaluated not only through radiographic outcomes but also through measures of patient experience, autonomy, and quality of life. Notably, while these broad patterns appear consistently across diverse patient populations and study designs, the magnitude and nature of their effects remain influenced by factors such as fracture location, duration of immobilization, patient age, and underlying comorbidities, underscoring the importance of individualized approaches to fracture management.

## 4. Discussion

### 4.1. Emerging Alternatives to Traditional Orthopaedic Casting

Traditional casting imposes burdens that extend across physical, psychological, emotional, social, and economic domains, highlighting the need for immobilization approaches that maintain fracture stability while improving patient experience. Advances in materials science and digital fabrication have produced emerging strategies, including breathable mesh systems, light-cured polymer mesh (LCPM) technologies, and customized 3D-printed orthoses [3,79]. These innovations seek to address common limitations of plaster and fiberglass casting, such as discomfort, hygiene challenges, and restricted mobility, while reflecting a broader shift toward patient-centered fracture care.

The evidence reviewed in this manuscript further suggests that successful fracture management should be evaluated using both structural and patient-centered outcomes rather than radiographic healing alone. Rather, immobilization represents a multidimensional patient experience that may simultaneously influence physical recovery, psychological well-being, emotional health, social participation, caregiver burden, and economic stability. Although traditional orthopaedic literature has historically emphasized radiographic healing and structural stability, the findings reviewed here demonstrate that patient-centered outcomes are also clinically meaningful determinants of recovery quality and overall treatment success. Importantly, many of these domains appear interconnected, as physical discomfort and functional restriction may contribute to psychological distress, social withdrawal, caregiver dependence, and downstream economic burden. This broader perspective supports the growing need for immobilization strategies that balance fracture stability with holistic patient-centered care principles, which may help explain the increasing clinical interest in emerging technologies such as LCPM systems and 3D-printed immobilizers.

Comparative studies have evaluated 3D-printed and polymer-based orthoses. Importantly, although both technologies demonstrate similar patient-centered advantages, their supporting evidence remains heterogeneous, with most studies consisting of feasibility investigations, small clinical cohorts, or early stage clinical evaluations. These findings should be interpreted within the broader context of multidimensional patient outcomes rather than solely as measures of structural success. However, much of the available evidence is derived from small-scale or early phase studies with limited follow-up. In stable, non-displaced upper-extremity fractures, emerging immobilization systems have demonstrated fracture union rates comparable to traditional casting, often exceeding 90% in selected cohorts, while also producing high patient satisfaction scores (approximately 85–95%) and improved patient-reported outcomes related to comfort, hygiene, and overall treatment experience [79,80,81,82,83]. These features have also been associated with lower rates of skin irritation during immobilization [3]. In selected fracture populations, earlier return to light functional activity, typically by approximately 1–2 weeks, has also been reported. Although emerging immobilization technologies demonstrated promising improvements in comfort, ventilation, hygiene, and patient satisfaction, direct evidence supporting superior outcomes related to psychological distress, social stigma, claustrophobia, or long-term functional stiffness remains limited [3,79,80,81,82,83]. Many proposed psychosocial benefits are currently inferred from improved usability and patient experience rather than established through large-scale comparative investigations [3,79,80,81,82,83]. Accordingly, these findings should be interpreted cautiously until further fracture-specific and population-specific studies are available [79,80,81,82,83].

The authors emphasize that emerging immobilization strategies should presently be viewed as complementary approaches rather than replacements for traditional casting methods. Established plaster and fiberglass techniques remain highly effective for fracture stabilization, and the available evidence primarily supports potential improvements in patient-centered outcomes rather than definitive superiority in clinical effectiveness. From the authors’ perspective, the greatest contribution of emerging immobilization technologies may not be their ability to improve fracture union, which already remains high with traditional methods, but rather their potential to reduce the physical, psychological, and social burdens that accompany prolonged immobilization. This shift reflects a broader evolution within orthopaedic practice toward value-based and patient-centered care, where treatment success is increasingly defined by patient experience and functional recovery in addition to structural healing. A comparison of traditional casting and emerging immobilization technologies is presented in Table 2.

Collectively, these findings suggest that the future value of immobilization technologies may lie not only in their ability to maintain fracture alignment, but also in their capacity to minimize the broader physical, psychological, and socioeconomic burdens traditionally associated with casting.

These developments reflect a broader shift toward patient-centered fracture care, with emerging studies consistently reporting improvements in comfort, hygiene, and overall treatment satisfaction while maintaining acceptable fracture stability in appropriately selected populations [79,80,81,82,83]. At the same time, important uncertainties remain regarding long-term functional outcomes, psychological benefits, and applicability across diverse fracture types and patient populations, as much of the current evidence derives from feasibility studies, small clinical cohorts, or investigations with limited follow-up durations [79,80,81,82,83].

Emerging immobilization systems are increasingly designed to balance fracture stability with usability and patient experience. Early studies suggest they may improve compliance and could potentially reduce selected complications, although larger comparative investigations are needed to confirm these findings [82]. LCPM systems have gained attention because their lightweight, ventilated design may improve patient experience while maintaining fracture stability in appropriately selected populations. These systems offer an open-lattice structure that promotes airflow and moisture evaporation, as well as reduced weight compared with traditional casting materials. Preliminary evaluations have demonstrated reductions in skin irritation and improvements in comfort and hygiene compared with circumferential casting approaches [83].

Despite these promising findings, several barriers to widespread adoption remain. These include higher upfront costs, the need for specialized equipment, and variability in access to digital fabrication technologies across healthcare settings [84]. In addition, much of the current evidence is derived from small-scale studies with limited follow-up durations, which may restrict generalizability and long-term conclusions. As a result, further large-scale comparative and cost-effectiveness studies are needed to more fully evaluate the clinical and economic impact of these emerging immobilization strategies.

### 4.2. Role of LCPM Systems in Addressing Casting Challenges

The limitations of traditional plaster and fiberglass casting extend across physical, psychological, emotional, social, and economic domains. In response to these challenges, LCPM systems have been developed with the goal of maintaining fracture stability while improving patient experience [1,3,83]. Early clinical studies have evaluated ventilated and lattice-style systems. In stable upper-extremity fractures, these systems maintain alignment in over 90% of cases. Rates of secondary displacement are low when they are applied correctly [79,83].

Preliminary observational studies and feasibility trials evaluating polymer mesh immobilizers have reported patient satisfaction rates ranging from approximately 85–95%, particularly in domains related to comfort, hygiene, and weight [83]. Skin irritation rates in these systems are generally low. Reported rates range from below 5–8%, compared to 5–15% in traditional casting. However, direct head-to-head comparisons are still limited [3,83]. Much of the available evidence is derived from small clinical studies with short-term follow-up, which may limit generalizability and the ability to draw conclusions regarding long-term outcomes [79,83]. Table 3 summarizes the major patient-centered outcomes associated with LCPM systems across physical, psychological, emotional, social, and economic domains. Although evidence remains preliminary, these findings illustrate how polymer-based and lattice-style immobilization approaches may address several limitations of conventional casting.

In our view, the promise of LCPM systems lies in their ability to address multiple patient-centered outcomes simultaneously rather than optimizing any single clinical endpoint. However, enthusiasm for these technologies should remain balanced by recognition that long-term effectiveness, cost-efficiency, and broad applicability across diverse fracture populations remain incompletely understood.

#### 4.2.1. Physical Benefits

LCPM systems use an open-lattice structure. This allows airflow and moisture to evaporate. These features are associated with reduced skin irritation, itching, and odor compared to traditional casts [1,3,83]. Experimental studies have evaluated humidity levels in these systems. They show reductions in localized moisture buildup. Reported decreases are about 20–40% compared to traditional plaster casts [3,83]. These systems are also typically 30–50% lighter than traditional plaster constructs, which may contribute to improved comfort during prolonged use [1,3,83].

The conforming design may allow for more even pressure distribution across the limb, which has been associated with reduced risk of pressure-related complications [3,83]. In addition, the waterproof properties of these materials enable routine hygiene practices without compromising structural integrity [1,3,83]. In small comparative cohorts, breathable polymer systems have demonstrated lower rates of dermatological complications relative to traditional casting, although larger comparative trials are needed to confirm these findings [3,83]. Light-activated curing times are typically under 5 min, allowing rapid stabilization while preserving the ability to adjust fit during application [83].

#### 4.2.2. Psychological Benefits

The removal process associated with polymer-based immobilization systems differs from traditional casting, as it does not require oscillating saws [85]. This eliminates the exposure to noise and vibration that has been associated with anxiety and distress, particularly in pediatric populations [85,86]. Some studies have looked at anxiety during cast removal. Systems that do not require saws show lower anxiety scores. Reported reductions range from about 40–70%, although data are still limited [85,87]. Qualitative reports have indicated that perceived control during removal may contribute to improved patient experience [87]. These findings suggest that the removal method may influence psychological responses during treatment, although further controlled studies are needed [85,87].

#### 4.2.3. Emotional Benefits

Improved comfort and usability associated with ventilated immobilization systems may support greater independence during recovery [88]. These systems allow patients to maintain hygiene more easily. They also support routine self-care activities. Small studies report increases in autonomy scores of about 15–30% [88]. In pediatric and adolescent populations, customization options such as color and design have been associated with increased satisfaction and reduced embarrassment related to cast wear [89,90]. Survey-based studies have reported improvements in perceived satisfaction and reductions in appearance-related distress in approximately 50–70% of pediatric patients when customization is available [89,90].

#### 4.2.4. Social Benefits

The reduced bulk and improved comfort of ventilated immobilization systems may facilitate participation in daily, occupational, and recreational activities [1,3,83]. Comparative studies have reported earlier return to light functional activity by approximately 1–2 weeks in selected fracture populations using removable or ventilated orthoses [79,91]. Improved comfort and mobility have also been associated with higher social participation scores during immobilization [91]. In pediatric populations, preliminary data suggest reductions in school absenteeism of approximately 2–5 days in selected cohorts [92], although broader validation is needed.

#### 4.2.5. Economic Benefits

LCPM systems are associated with higher upfront material costs compared with traditional plaster or fiberglass casting, with reported costs approximately 2–4 times greater depending on setting and production methods [1,3,83]. However, preliminary evidence suggests that these systems may have the potential to reduce downstream costs through fewer unplanned cast changes and lower complication rates, although formal cost-effectiveness analyses remain limited [93]. In feasibility studies, unplanned cast replacement rates have been reported below 3–5% in ventilated systems, compared with 5–15% in traditional casting populations [3,93]. Earlier return to daily activities may also contribute to reductions in indirect costs, including lost productivity and caregiver burden [6,70,93]. Economic models have evaluated these systems. Reducing work absence by even 1–2 weeks may help offset higher initial costs. However, formal cost-effectiveness data are still limited [93].

### 4.3. Study Limitations

This narrative review has several limitations. Although a structured and transparent approach was used, it does not meet the criteria of a formal systematic review. This creates potential for selection bias, and some relevant studies may not have been included. The included literature is also heterogeneous. In addition, fracture-specific and patient-specific variables may substantially influence both complication rates and patient-reported outcomes [31,32,33,34,35,36,37,38,39,40,41,42]. Included studies varied considerably with respect to anatomical region (upper versus lower extremity), fracture type and severity, duration of immobilization, adjacent joint fixation strategies, patient age, comorbidities such as diabetes or vascular disease, and socioeconomic context [31,32,33,34,35,36,37,38,39,40,41,42,61,71,78]. Because these factors were inconsistently reported across the literature, subgroup-specific synthesis and direct comparison between traditional casting, LCPM systems, and 3D-printed immobilization approaches were limited. Accordingly, conclusions regarding comparative effectiveness should be interpreted cautiously until more standardized fracture-specific investigations become available.

Importantly, direct comparisons among traditional casting, LCPM systems, and 3D-printed orthoses should be interpreted with caution, as available evidence derives from heterogeneous study designs, fracture populations, clinical settings, and outcome measures. Much of the literature concerning emerging technologies consists of feasibility studies, pilot investigations, or small observational cohorts rather than large-scale comparative trials. Consequently, observations regarding potential advantages should be considered hypothesis-generating rather than definitive evidence of superiority.

In addition, publication bias and selective outcome reporting may influence interpretation of the available evidence, particularly regarding emerging immobilization technologies [3,61,71,78]. Positive findings related to patient satisfaction, comfort, and usability may be more likely to be published than neutral or unfavorable results. Furthermore, many investigations involving LCPM and 3D-printed systems remain preliminary, industry-associated, or limited by small sample sizes and short follow-up durations, which may affect generalizability and increase the risk of overestimating potential benefits [61,71,78]. Much of the evidence on emerging technologies, including LCPM and 3D-printed systems, comes from small studies with short follow-up. This limits the ability to assess long-term outcomes, complication rates, and cost-effectiveness. Direct comparisons with traditional casting are also limited. Finally, there is potential for interpretive bias, especially when combining qualitative and patient-reported data. Although efforts were made to remain balanced, the focus on patient-centered outcomes and newer technologies may reflect current trends in orthopaedic research.

The authors believe that future investigations should prioritize standardized patient-reported outcome measures, fracture-specific analyses, and longer follow-up periods to better determine whether emerging technologies provide durable advantages beyond short-term improvements in comfort and usability.

## 5. Conclusions

Traditional orthopaedic casting remains effective for fracture stabilization but is associated with multidimensional patient burden that extends beyond bone healing. The central finding of this review is that fracture immobilization should be conceptualized as a multidimensional component of patient care rather than solely a mechanical intervention. Technologies that simultaneously preserve fracture stability and improve patient experience may ultimately redefine how treatment success is evaluated within orthopaedic practice. These challenges are often more pronounced in vulnerable populations and underscore the need for more patient-centered approaches. Emerging alternatives, including LCPM and 3D-printed immobilizers, demonstrate promising potential to address several limitations associated with traditional casting, particularly in domains related to comfort, hygiene, usability, and patient experience, while preliminary evidence suggests maintenance of fracture stability in selected populations. Although initial costs may be higher, potential reductions in complications, follow-up visits, and caregiver burden may offer long-term value in selected clinical settings. Future research should prioritize large-scale, fracture-specific comparative investigations incorporating standardized patient-reported outcome measures, long-term functional assessments, healthcare utilization metrics, and formal cost-effectiveness analyses across diverse patient populations.

Importantly, variability in fracture type, anatomical region, immobilization strategy, patient comorbidities, and socioeconomic context across the available literature currently limits broad generalization of comparative findings between traditional casting, LCPM systems, and 3D-printed immobilization approaches. Fracture care should not be evaluated solely on radiographic healing but should also account for patient dignity, independence, and overall well-being. In addition, continued advances in material science, digital fabrication, and personalized orthopaedic design may further accelerate the transition toward individualized and patient-centered immobilization strategies. As fracture care continues to evolve, future immobilization approaches may increasingly prioritize comfort, usability, psychological well-being, and overall recovery experience alongside traditional biomechanical goals.

Looking forward, the integration of advanced materials, digital fabrication techniques, and patient-reported outcome measures may fundamentally reshape how immobilization success is defined within orthopaedic practice. Future fracture care models may increasingly prioritize individualized treatment strategies that account not only for anatomical healing but also for psychological well-being, functional independence, social participation, and economic considerations. In this context, emerging immobilization technologies should be viewed not merely as alternative devices, but as components of a broader movement toward holistic, value-based, and patient-centered musculoskeletal care. Collectively, these findings support a shift toward immobilization strategies that balance structural outcomes with multidimensional patient-centered impact.

## Figures and Tables

**Table 1:** Multidimensional Impacts of Traditional Orthopaedic Casting.

Domain	Reported Impacts	Representative Findings	Key References
Physical	Joint stiffness, muscle atrophy, skin irritation, pressure sores, hygiene difficulties	Immobilization is associated with reduced joint ROM, muscle wasting, dermatologic complications, and challenges maintaining skin integrity, particularly with prolonged casting	[3,4,5,6]
Psychological	Anxiety, claustrophobia, fear of cast removal, depressive symptoms	Cast-induced anxiety and claustrophobia are commonly reported, with oscillating saw removal identified as a major source of distress, especially in pediatric populations	[4,34,35,36]
Emotional	Loss of autonomy, frustration, diminished self-esteem	Dependence on caregivers and inability to perform activities of daily living contribute to emotional distress and reduced self-efficacy during recovery	[5,37,38,39]
Social	Reduced participation, social withdrawal, caregiver strain	Casting frequently limits school, work, and social engagement; caregiver burden and altered family dynamics are common, particularly in pediatric and geriatric cases	[6,48,49,50]
Economic	Out-of-pocket expenses, lost wages, caregiver productivity loss	Despite low material costs, traditional casts generate substantial indirect costs, including absenteeism, caregiver time, transportation, and repeat clinical visits	[39,40,41,42,43,44,45,46,47]

**Table 2:** Comparison of Traditional Casting and Emerging Immobilization Technologies.

Feature/Outcome Domain	Plaster & Fiberglass Casts	FlexiOH Light-Cured Polymer System	3D-Printed Lattice Immobilizers	Representative References
Structural stability	Well-established fracture stabilization when properly applied	Provides rigid immobilization comparable to traditional casts after curing	Custom-fit rigidity with reinforcement of stress zones	[1,2,3,30]
Weight and bulk	Relatively heavy and bulky, contributing to fatigue and movement restriction	Lightweight, low-profile design reduces limb fatigue	Lightweight and anatomically contoured	[3,30]
Ventilation and hygiene	Poor ventilation; moisture retention and odor common	Open-lattice mesh allows airflow, evaporation, and washability	Open lattice improves airflow and hygiene	[3,42,43]
Skin and soft-tissue complications	Higher rates of skin irritation, maceration, and pressure-related injury	Reduced skin irritation and improved moisture control	Reduced skin contact; lower risk of maceration	[3,4,5,6,30]
Psychological impact	Claustrophobia, anxiety, and distress during wear and removal; saw-related fear common	Quiet, saw-free removal reduces anxiety and anticipatory distress	Removal generally less distressing than saw-based techniques	[34,35,36]
Emotional and social impact	Reduced autonomy, activity restriction, and social withdrawal	Improved comfort and usability support autonomy and social participation	Improved aesthetics and fit may support patient satisfaction and perceived usability	[37,38,39,40,41,42,43,44,45]
Patient convenience	Not waterproof; hygiene and daily activities limited	Waterproof and washable; supports daily living activities	Often removable for hygiene depending on design	[3,42]
Economic considerations	Low upfront material cost but substantial indirect and downstream costs	Higher initial cost with the potential to reduce selected downstream healthcare utilization, although formal cost-effectiveness evidence remains limited	Higher production cost; cost-effectiveness evolving	[39,40,41,42,43,44,45,46,47]
Access and scalability	Widely available, minimal equipment required	Requires curing light and trained personnel	Requires scanning, design software, and fabrication	[30,48,49,50]
Current evidence base	Extensive historical and contemporary literature	Growing clinical and patient-reported outcome evidence	Early stage and pilot clinical studies	[3,30]

**Table 3:** Patient-Centered Outcomes Associated with Light-cured Polymer Mesh Immobilization Systems (LCPM).

Outcome Domain	Patient-Centered Measure	Reported Findings with LCPM	Clinical Relevance	Representative References
Comfort	Wear tolerance, perceived bulk, thermal comfort	Patients consistently report improved comfort due to lightweight design, reduced bulk, and enhanced ventilation compared with traditional casts	Improved comfort may enhance adherence to immobilization and reduce premature cast removal	[3,30]
Skin integrity & hygiene	Itching, odor, maceration, ability to bathe	Open-lattice, washable design supports hygiene, reduces moisture accumulation, and lowers risk of skin irritation and odor	Reduced dermatologic complications may decrease unplanned visits and recasting	[3,4,5,6,42,43]
Psychological response	Anxiety, claustrophobia, fear of removal	Absence of oscillating saw during removal substantially reduces anxiety, particularly in pediatric and anxious patients	Lower anxiety may contribute to improved treatment experience and trust in care	[31,32,33,34,35,36]
Autonomy & independence	Ability to perform ADLs, self-care	Waterproof and lightweight construction allows greater independence in bathing and daily activities	Preservation of autonomy supports emotional well-being and recovery	[37,38,39]
Social participation	School attendance, work participation, social engagement	Improved usability and reduced stigma support continued participation in school, work, and social activities	Social engagement is linked to better psychological and functional outcomes	[31,45]
Caregiver burden	Time demands, stress, assistance required	Reduced hygiene challenges and fewer complications may lessen caregiver workload, particularly in pediatric and geriatric cases	Lower caregiver burden improves family functioning and adherence to care	[46,47,48,49]
Treatment satisfaction	Overall satisfaction, preference vs. traditional casts	Early reports indicate favorable patient and caregiver satisfaction, although direct comparative evidence with traditional casting remains limited	Satisfaction influences compliance and acceptance of emerging technologies	[30]
Economic implications (indirect)	Missed work/school, repeat visits	Improved durability and the potential for fewer unplanned interventions may decrease indirect costs despite higher initial material expense, although formal economic evaluations remain limited.	Indirect cost reduction is critical for value-based care models	[39,40,41,42,43,44,45,46,47]

## Data Availability

No new data were created or analyzed in this study.

## Abbreviations

ADL	Activities of Daily Living
EQ-5D	EuroQol 5-Dimension Questionnaire
LCPM	Light-Cured Polymer Mesh
MeSH	Medical Subject Headings
PoP	Plaster of Paris
PRISMA	Preferred Reporting Items for Systematic Reviews and Meta-Analyses
RCT	Randomized Controlled Trial
SF-36	Short Form (36) Health Survey
